# Preparation and Performance of Foam Lightweight Soil Synergistically Modified by Aeolian Sand and Oil Sludge Pyrolysis Residue for Desert Applications

**DOI:** 10.3390/ma19122527

**Published:** 2026-06-11

**Authors:** Bin Wang, Kaiyuan Wang, Jie Liu, Zheng Lu, Keqi Ren, Shiyu Zhu

**Affiliations:** 1 Xinjiang Transportation Science Research Institute Co., Ltd., Urumqi 830006, China; wangbin13245@126.com (B.W.);; 2 College of Transportation and Logistics Engineering, Xinjiang Agricultural University, Urumqi 830006, China; 3 Institute of Rock and Soil Mechanics, Chinese Academy of Sciences, Wuhan 430071, China; 4 College of Water Resources and Architectural Engineering, Shihezi University, Shihezi 832003, China; rekeqi@163.com; 5 College of Civil Engineering, Anhui Jianzhu University, Hefei 230601, China

**Keywords:** foam lightweight soil, aeolian sand, oil sludge pyrolysis residue, physical properties, mechanical properties, durability characteristics

## Abstract

The scarcity of natural aggregates and the accumulation of oil sludge in desert regions pose critical challenges for highway construction. Although aeolian sand and oil sludge pyrolysis residue have been studied individually as construction materials, their combined use in foamed lightweight soil remains unexplored. This study addresses this gap by developing a novel foamed lightweight soil termed SOFS, which is created through the synergistic modification of aeolian sand and oil sludge pyrolysis residue. A six-factor, five-level orthogonal array (L25) was employed to systematically investigate the effects of residue content, sand content, foam-to-slurry ratio, foaming agent dilution, water-to-solid ratio, and mixing time. The evaluated properties included physical properties (fluidity and wet density), mechanical properties (compressive, splitting tensile, and flexural strength), and durability (wet–dry and freeze–thaw resistance). Scanning electron microscopy was used to examine the microstructural mechanisms. Variance and range analysis identified the optimal mixture, designated H14, which achieved 28-day compressive, splitting tensile, and flexural strengths of 3.75 MPa, 2.21 MPa, and 0.9 MPa, respectively, thereby meeting desert roadbed requirements. Compared with conventional materials, H14 exhibited superior durability, with strength losses of only 16.3% in compressive strength and 19.1% in splitting tensile strength after 25 cycles. Microstructural analysis revealed a dense C-S-H gel network encapsulating the solid waste particles, with nanoscale Al- and Cl-rich crystalline phases observed at interfacial pores—a phenomenon that has rarely been documented in previous studies. These findings provide a theoretical and technical foundation for solid waste valorization and the development of sustainable desert infrastructure.

## 1. Introduction

SOFS (aeolian sand-oil sludge pyrolysis residue foamed lightweight soil) is a composite lightweight fill material produced by blending desert aeolian sand with oil sludge pyrolysis residue, cementitious agents, water, and pre-formed foam. It is primarily applied in backfill engineering, subgrade construction, and foundation treatment. Its key advantages are twofold: environmental sustainability, achieved through the valorization of solid wastes, and enhanced engineering performance, particularly its low self-weight, which helps reduce overburden pressure.

The construction of highways in desert regions, particularly in the hinterland of the Taklamakan Desert in Xinjiang, is confronted with critical technical and environ-mental challenges. With the advancement of China’s “Belt and Road” initiative, it is projected that the total length of newly constructed desert highways in Xinjiang could approach 2000 km by 2025 [1,2]. However, three interrelated bottlenecks hinder progress: (1) the prohibitively high transportation costs of conventional construction materials, which account for over 45% of total project expenditures; (2) ecological degradation in the Tarim River Basin, exacerbated by excessive river sand extraction; and (3) the markedly reduced performance of conventional road base materials under extreme desert environmental conditions [3,4]. Moreover, the scarcity of qualified natural aggregate resources along desert highway routes underscores the urgent need for in situ resource utilization technologies. Consequently, the substitution of traditional construction materials with locally available solid wastes has emerged as a critical research priority.

**Figure 1:**
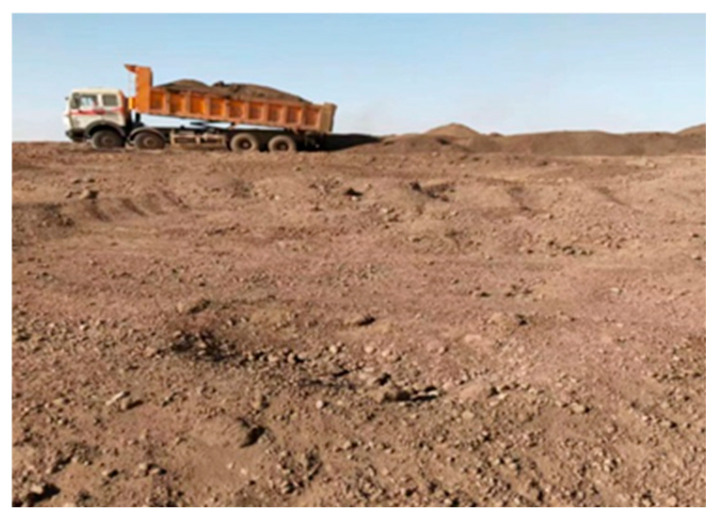
Aeolian Sand.

**Figure 2:**
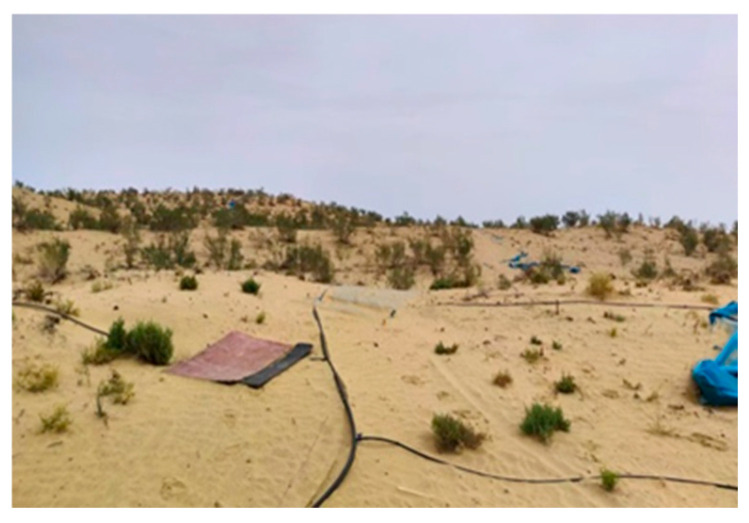
Oil Sludge Pyrolysis Residue.

Among the locally available resources in Xinjiang’s desert oil-producing regions, substantial quantities of oil sludge pyrolysis residue and aeolian sand have accumulated (Figure 1 and Figure 2). Oil sludge is primarily generated from container cleaning operations and contaminated soil during petroleum extraction [5]. Owing to its high hydrocarbon content, direct utilization is infeasible, and prolonged open-air storage poses considerable environmental hazards. Although pyrolysis has proven effective for treating such solid waste, large volumes of residue continue to ac-cumulate in stockpiles. Aeolian sand, formed through wind-induced transport and deposition, is widely distributed yet remains largely underutilized [6]. The efficient synergistic utilization of these two resources thus represents a pivotal step toward overcoming the technical barriers impeding desert highway construction.

Significant progress has been made in the individual use of these materials for engineering applications [7]. Ran et al. [8] demonstrated that composite modification of oil sludge pyrolysis residue enhances the rutting factor of asphalt mortar by 19.12%. In a related study, they reported that sandy subgrade specimens containing 25% residue achieved a 14-day unconfined compressive strength exceeding 0.4 MPa [9]. Gao [10] identified an optimal cement content of 4% for subgrade materials incorporating oil sludge pyrolysis residue, and this dosage was consequently adopted as the baseline cement content for all SOFS mixtures in the present study. Parallel investigations on aeolian sand have confirmed its technical viability for road construction. Khan et al. [11] first systematically demonstrated the feasibility of using Sahara Desert aeolian sand in arid environments. Through a cross-regional comparative study, Padmakumar [12] confirmed its suitability as a fine aggregate in concrete. Al-Harthy et al. [13] further reported that while replacing conventional fine aggregates with aeolian sand improved concrete workability, water absorption increased proportionally with the replacement ratio. Although these studies collectively confirm the engineering applicability of both materials, two critical gaps persist: (1) a systematic approach to controlling differential settlement in soft desert subgrades and aeolian sand foundations remains undeveloped, and (2) the long-term performance evolution under coupled material–structure–environment interactions is not yet understood.

Foamed lightweight soils offer a potential solution for addressing differential settlement due to their low density, high strength, plasticity, and self-supporting characteristics [14]. However, these materials are costly and can develop internal stresses during service. Such internal stresses arise from multiple mechanisms. First, the introduction of air bubbles disrupts slurry equilibrium, generating localized stress fields through interactions between bubble surface tension and slurry viscosity [15]. Second, during hardening, heat release and shrinkage from cement hydration, combined with the presence of bubbles, induce uneven heat transfer and create microscopic tensile and compressive stresses around pore walls [16]. Third, under external loading, bubbles act as defect sites, leading to stress concentration [17]. These internal stress mechanisms are key microscale factors that influence long-term durability and mechanical behavior, representing a critical direction for investigation.

Recent efforts to reduce both cost and internal stresses have focused on incorporating external admixtures into foamed lightweight soils. Alharthai et al. [18] employed palm oil fuel ash, rice husk ash, and bamboo leaf ash as partial cement substitutes. They confirmed that the mixtures complied with masonry strength requirements. Riyap et al. [19] showed that compressive strength development in rice husk-modified metakaolin systems was governed primarily by the synergistic effects of cement content and fiber addition. Xiao et al. [20] reported that incorporating recycled concrete powder reduced thermal conductivity by 23.7% and compressive strength by 18.4%. This approach also expands opportunities for construction waste valorization. Jin et al. [21] enhanced the mechanical properties and durability of foamed lightweight soils through a multifunctional strategy involving natural fiber combinations and optimization of the foam-to-slurry volume ratio. Wu et al. [22] developed polypropylene fiber-reinforced foamed lightweight soil, confirming significantly improved resistance to wet–dry cycling. Selija et al. [23] developed a dual-source natural foaming system utilizing sesame seed protein and Indian mangosteen saponin, successfully preparing eco-friendly foamed lightweight soils and establishing optimal water-to-solid and foaming agent ratios.

Previous studies have explored modification mechanisms using various admixtures, including natural fibers, industrial solid wastes, and bio-based foaming agents. Nevertheless, two major limitations persist. First, most research has focused on optimizing individual admixtures, with limited attention given to the synergistic effects of combined solid waste utilization. Second, the challenges posed by specific geographical contexts, such as aggregate scarcity and high transportation costs in desert regions, remain insufficiently addressed. Furthermore, the particle size characteristics of aeolian sand and oil sludge pyrolysis residue meet the technical requirements for use as admixtures in foamed lightweight soil. However, the interfacial effects, long-term durability, and environmental benefits of their combined application have yet to be systematically investigated.

To address these gaps, the present study aims to develop a lightweight foamed soil suitable for subgrade filling by incorporating aeolian sand and oil sludge pyrolysis residue as complementary admixtures. Both materials—as naturally available resources and solid wastes with particle sizes below 5 mm—meet the fundamental criteria for auxiliary materials in foamed lightweight soil, offering the potential to simultaneously reduce production costs and mitigate internal stresses. An orthogonal experimental design based on the L25(5^6^) array was employed to evaluate the main effects of six factors: the dosages of aeolian sand and oil sludge pyrolysis residue, the water-to-solid ratio, the foam-to-slurry volume ratio, the foaming agent dilution ratio, and the mixing time. This approach enables systematic evaluation using only 25 experimental runs, leveraging the uniform dispersion property of orthogonal designs. Subsequently, the physical properties (fluidity and wet density) and mechanical properties (unconfined compressive strength, splitting tensile strength, and flexural strength) were evaluated to identify the optimal formulation. Durability performance was verified under simulated desert conditions, and the underlying microstructural mechanisms were elucidated. The findings aim to advance the effective recycling of solid waste resources and provide sustainable solutions for desert highway development.

## 2. Test Materials and Methods

For each experimental condition, five identical specimens were prepared and tested to ensure reproducibility. All measurements were performed in triplicate, and the average values are reported.

### 2.1. Raw Materials

#### 2.1.1. Cement

Ordinary Portland cement (P.O 42.5, Tianshan Cement Co., Ltd., Urumqi, China) was used in this study, and its main technical specifications are shown in Table 1.

#### 2.1.2. Aeolian Sand

**Figure 3:**
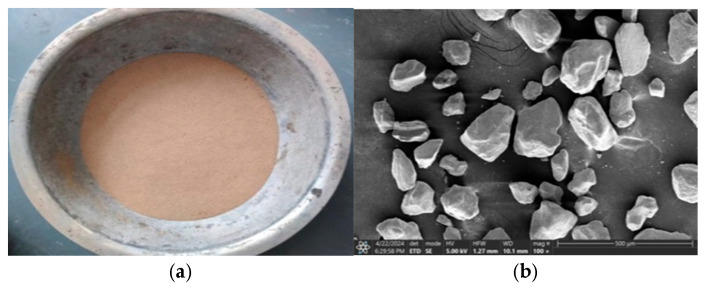
(**a**) Aeolian Sand, (**b**) SEM of Aeolian Sand.

The aeolian sand used in this study was collected from the Gurbantunggut Desert in Xinjiang, China (Figure 3). Its coefficient of uniformity (Cu) is 4, and its coefficient of curvature (Cc) is 1. The physical and chemical properties of the aeolian sand are presented in Table 2.

#### 2.1.3. Oil Sludge Pyrolysis Residue

**Figure 4:**
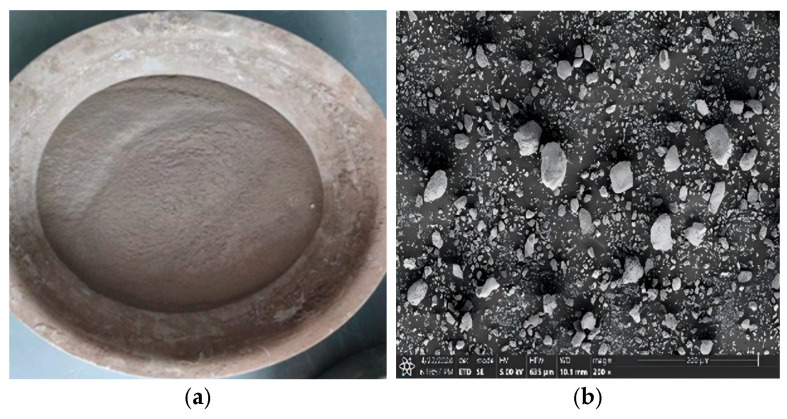
Aeolian Sand and Oil Sludge Pyrolysis Residue. (**a**) Oil Sludge Pyrolysis Residue, (**b**) SEM of Oil Sludge Pyrolysis Residue.

The oil sludge pyrolysis residue used in this study was sourced from Luntai County, Bayingolin Mongol Autonomous Prefecture, Xinjiang, China. Following mechanical abrasion treatment, the residue was in powdered form (Figure 4a), while it exhibited a flocculent morphology after drying (Figure 4b). The particle size distributions of both the aeolian sand and the oil sludge pyrolysis residue are summarized in Table 3. Based on standard geotechnical classification criteria, gradation analysis indicates that both materials are characterized as poorly graded.

#### 2.1.4. Foaming Agent Type

According to the Technical Specification for Design and Construction of Cast-in-situ Foamed Lightweight Soil Subgrade (TJGF1001-2011) [24] and previous studies [15], the recommended foaming agent dilution ratio ranges from 35 to 55. Preliminary experimental results indicated that the HT composite foaming agent exhibited superior performance compared to the HY composite foaming agent, the resin-based foaming agent, and the AOS foaming agent in terms of key indicators such as wet density, water secretion rate, and foaming multiple. Based on these advantages, the HT composite foaming agent was selected for use in this study, and its main performance parameters are presented in Table 4.

### 2.2. Test Methods

#### 2.2.1. Physical Characterization

The flow test was conducted following the vertical cylinder method in accordance with ASTM C230/C230M [25] and ASTM C1437 [26]. A standard cylinder was placed at the center of a glass plate and filled with slurry until slight overflow occurred. The cylinder was then lifted vertically at a constant speed to allow the slurry to spread. After 60 s, the maximum diameter of the spread slurry was measured in two perpendicular directions, and the average value was recorded. Wet density was determined using the mass difference method per ASTM C185 [27]. A clean, dry 1 L volumetric cylinder was weighed and filled with slurry in layers to the overflow point, and the surface was leveled by scraping to remove any excess. The cylinder containing the slurry was weighed twice, and the wet density was calculated using Equation (1), with results rounded to the nearest 0.1 kg/m^3^.

Where m_1_ is the mass of the volumetric cylinder (g), m_2_ is the mass of the volumetric cylinder and the specimen (g), V is the volume of the volumetric cylinder (L), and ρ_w_ is the wet density (kg/m^3^),

(1) $$\rho_{w}=\frac{m_{2}-m_{1}}{V}$$

#### 2.2.2. Mechanical Performance Test

Specimens measuring 100 mm × 100 mm × 100 mm were prepared for unconfined compressive strength and splitting tensile strength tests, while specimens of 100 mm × 100 mm × 400 mm were cast for flexural strength testing according to the designated mix proportions. After 28 days of standard curing, mechanical properties were measured using a WAW-1000B electro-hydraulic universal testing machine (Changchun Testing Machine Company, Changchun, China). In accordance with ASTM C39 [28], C496 [29], and C78 [30], the loading rates were maintained at 0.5 MPa/s for the compressive and splitting tensile tests and 0.05 mm/s for the flexural tests. Failure was defined as the point at which the specimen could no longer sustain the applied load, characterized by a sudden drop in load capacity or visible fracture.

#### 2.2.3. Durability Test

The test was conducted in accordance with ASTM D4867/D4867M-2022 [31]. Cubic specimens (100 mm × 100 mm × 100 mm) were prepared for the wet–dry cycling test, with three specimens allocated to the parallel control group and three to the cyclic test group. All specimens were initially cured under standard conditions for 28 days. Specimens in the cyclic group were first dried at 60 ± 5 °C and then subjected to 25 cycles. Each cycle consisted of drying at 60 °C for 7 h, cooling at 20 °C for 20 min, immersion in water to a depth of 30 mm for 5 min, and drying for 30 min. The parallel control group specimens were continuously maintained under standard curing conditions for the same duration. After completing the cycles, the average splitting tensile strength of both groups was compared, and the wet–dry strength coefficient was calculated using Equation (2), with results reported to two decimal places.

Where K is dry and wet strength coefficient, $f_{ts}^{\prime }$ is average value of split tensile strength of cyclic group specimens in MPa, and $f_{ts}$ is average value of parallel group specimen split average value of tensile strength in MPa,

(2) $$K=\frac{f_{ts}^{\prime }}{f_{ts}}$$

The freeze–thaw cycle test was conducted on five groups of 100 mm^3^ cubic specimens (3 per cyclic/control group) that were prefabricated and cured for 28 days. The pre-treatment process involved “half-immersion (20 ± 2 °C for 24 h) → full immersion (top 30 mm for 24 h) → sealed static (24 h).” The cyclic group was subjected to freeze–thaw cycles in a freeze–thaw chamber, with a total of 25 cycles applied. Each cycle consisted of −15 ± 2 °C for 8 h, followed by 20 °C for 6 h with water thawing. The parallel control group specimens were stored under standard curing conditions during the same period. Afterward, specimens were gradient-dried (60 °C → 80 °C → 105 °C) to constant weight, and compressive strength was determined on *f*_1d_ and *f*_2d_. Frost resistance was assessed by the rate of strength loss (Equation (3)), with an accuracy of 0.1%, in a 20 ± 2 °C water environment.

Where *F*_m_ is the compressive strength loss as a percentage; *f*_1d_ is the compressive strength of the freeze–thaw specimens, in MPa; and *f*_2d_ is the compressive strength of the parallel specimens, in MPa, after the freeze–thaw test,

(3) $$F_{m}=\frac{f_{2d}-f_{1d}}{f_{2d}}\times 100\%$$

In arid regions, surface soils experience multiple wetting–drying alternations per year due to sporadic precipitation followed by rapid evaporation. Comparable durability studies on foamed lightweight soils for roadbeds typically adopt 12–20 cycles [22]. Given that the Taklamakan Desert is characterized by a hyperacid climate with less than 50 mm annual precipitation [32]—where extreme evaporation accelerates moisture loss, intensifying surface soil drying—this study adopted a more rigorous 25-cycle regime to ensure a conservative performance assessment.

#### 2.2.4. Micro Testing

Based on the results obtained from the physical, mechanical, and durability performance tests, five representative mix proportions were selected for microstructural observation, which was performed using a Sigma-300 scanning electron microscope (Carl Zeiss AG, Oberkochen, Germany).

### 2.3. Test Program

#### 2.3.1. General Research Program

**Figure 5:**
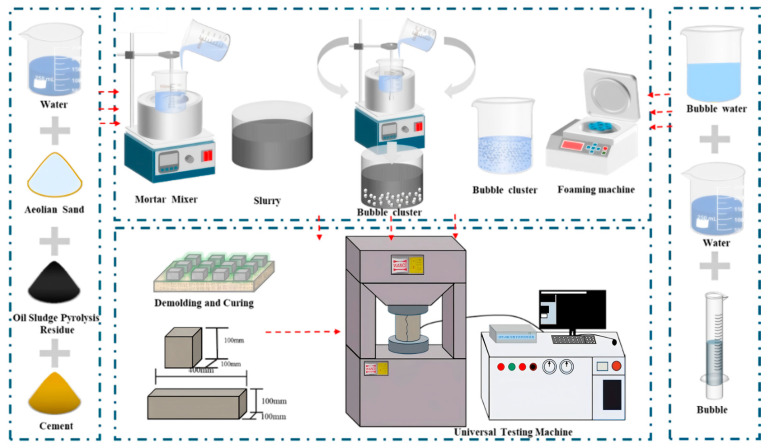
SOFS preparation flow.

The foaming liquid was initially prepared at the designated dilution ratio to produce stable foam. Subsequently, water, sand, cement, and pre-treated oil sludge residue were weighed and mixed with the foam to obtain a homogeneous slurry. The mixture was then injected into Vaseline-coated molds, leveled, and sealed. After pneumatic demolding, the specimens were cured for 28 days prior to testing, as shown in Figure 5.

**Figure 6:**
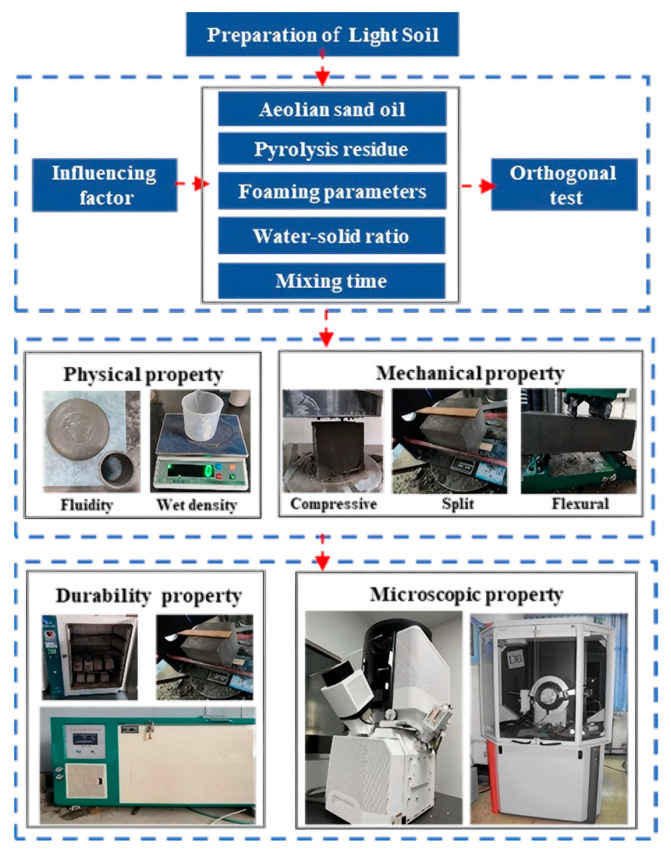
Flow chart of SOFS test.

The SOFS test flow in this paper is shown in Figure 6.

#### 2.3.2. Mixing Ratio Design

The dosage ranges for the key experimental factors were established based on a combination of preliminary trial mixes and specifications from established technical standards and literature. Specifically, the cement content was fixed with reference to the optimal dosage identified by Gao [10] for subgrade materials utilizing oil sludge pyrolysis residue. The ranges for the water-to-solid ratio (0.29:1 to 0.33:1) and foaming agent dilution ratio (35 to 55) were determined in accordance with the Technical Specification for Design and Construction of Cast-in-situ Foamed Lightweight Soil Subgrade (TJGF1001-2011) and the study by Liu et al. [16], ensuring an initial balance between foam stability and slurry workability. The upper limits for the incorporation of aeolian sand and oil sludge pyrolysis residue were informed by previous individual feasibility studies [8,9,13], which demonstrated acceptable mechanical performance at high replacement ratios. The specific levels for each factor, as detailed in Table 5, were subsequently refined through an orthogonal experimental design (L25 Taguchi array) to systematically evaluate their synergistic effects and identify the optimal combination.

In this study, the 28-day compressive strength, wet density, and fluidity were adopted as the primary control indices. The slurry performance was adjusted through coordinated control of foaming parameters and mixing time to maximize the solid waste substitution rate while meeting the specified requirements. The total solid mass was first calculated using Equation (4), followed by the water consumption via Equation (5). The foam mass was then determined using Equation (6), and the rationality of the mix proportion was verified with Equation (7). Finally, Equation (8) was used to convert foam mass to volumetric parameters for engineering applications. Here, mc, ma, mo, and ms denote the masses of cement, oily sludge, aeolian sand, and total solids, respectively; mw is the mass of water; W/S is the water-to-solid ratio of cement; mf is the foam mass; γ is the unit weight; and pc, pa, po, pw, and pf represent the densities of cement, oily sludge, aeolian sand, water, and foam, respectively.

(4) $$m_{s}=m_{c}+m_{a}+m_{o}$$

(5) mw=(WS)×ms

(6) $$m_{s}+m_{w}+m_{f}=100\gamma$$

(7) $$\frac{m_{c}}{\rho_{c}}+\frac{m_{a}}{\rho_{a}}+\frac{m_{o}}{\rho_{o}}+\frac{m_{w}}{\rho_{w}}+\frac{m_{f}}{\rho_{f}}=1$$

(8) $$V_{f}=1000\times \left[1-\left(\frac{m_{c}}{\rho_{c}}+\frac{m_{a}}{\rho_{a}}+\frac{m_{o}}{\rho_{o}}+\frac{m_{w}}{\rho_{w}}\right)\right]$$

Based on preliminary experiments and the Technical Specification for Design and Construction of Cast-in-situ Foamed Lightweight Soil Subgrade (TJGF1001-2011), the experimental factors and their ranges were established as follows: Factor A takes the values of 0, 60, 90, 120, and 150 g/dm^3^; Factor B takes the same values of 0, 60, 90, 120, and 150 g/dm^3^; Factor C ranges from 0.8:1 to 1.2:1 in increments of 0.1; Factor D ranges from 35 to 55 in increments of 5; Factor E ranges from 0.29:1 to 0.33:1 in increments of 0.01; and Factor F ranges from 80 to 120 s in increments of 10 s. The specific factor levels and the orthogonal test arrangements are presented in Table 5 and Table 6, respectively.

## 3. Results

### 3.1. SOFS Physical Properties

#### 3.1.1. Flowability

Based on the ANOVA results, Factor B had a highly significant effect on the outcome (*p* = 0.00093 < 0.05), while Factor E also showed a significant effect (*p* = 0.004 < 0.05). Factor A approached significance (*p* = 0.080), falling slightly above the conventional threshold, which suggests marginal significance that warrants further interpretation within the professional context. Factors C and D had no statistically significant effects, with *p*-values of 0.526 and 0.411, respectively. The F-values further confirmed that Factors A, B, and E significantly influenced the flowability of SOFS, as their values exceeded the corresponding critical thresholds: A (F = 2.375 > F0.1(4,25) = 2.18), B (F = 9.347 > F0.01(4,25) = 4.177), and E (F = 4.984 > F0.01(4,25) = 4.177). The remaining factors showed no significant influence within the tested confidence levels.

**Figure 7:**
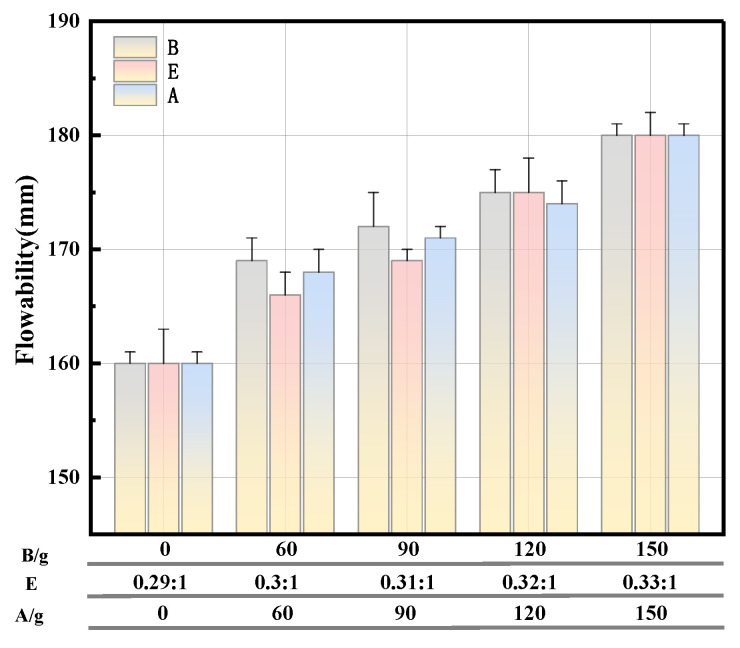
Effect of different factors on flowability.

As shown in Figure 7, increasing factor A (oil sludge residue content) from 0 to 150 g enhanced flowability from 160 mm to 175 mm. This improvement may be attributed to reduced interparticle cohesion resulting from the increased solid phase content, a phenomenon consistent with observations in coal gangue-modified foamed concrete [33]. Flowability also increased from 160 mm to 180 mm with increasing factor B (aeolian sand content), primarily due to the ball-bearing effect of its non-plastic particles, which reduces internal friction [13]. When factor E (water-to-solid ratio) was increased from 0.29:1 to 0.33:1, flowability improved notably from 160 mm to 179 mm. This trend aligns with the findings of Yang [34], who reported that increased water content promotes slurry diffusion through gravity-driven effects and reduces viscous resistance by thickening the free water film.

#### 3.1.2. Wet Density

**Figure 8:**
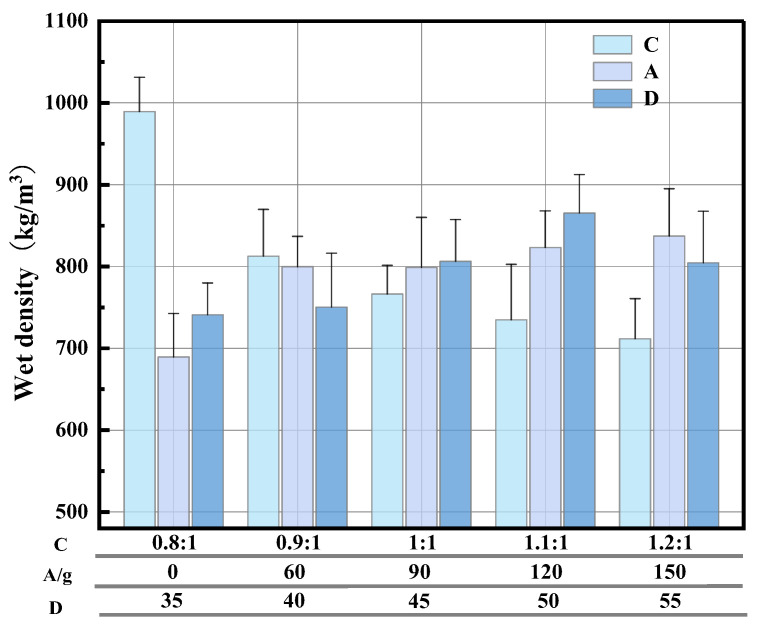
Effect of different factors on wet density.

ANOVA indicated that Factors A, C, and D had a significant effect on the wet density of SOFS: A (F = 2.47 > F0.1(4,25) = 2.18), C (F = 9.204 > F0.01(4,25) = 4.177), and D (F = 2.378 > F0.1(4,25) = 2.18). The remaining factors showed no significant influence. As illustrated in Figure 8, increasing Factor A from 0 to 150 g/dm^3^ across its five discrete levels, 0, 60, 90, 120 and 150, led to a 21.4% increase in wet density from 689.4 to 837.1 kg/m^3^, attributed to a reduction in porosity caused by particle shrinkage and water absorption. Increasing Factor C from 0.8:1 to 1.2:1 resulted in a 28.1% decrease in wet density (from 989.4 to 711.7 kg/m^3^), possibly due to the dilution of solid components by the increased foam content. Factor D exhibited a nonlinear effect on wet density, with the maximum value (785.3 kg/m^3^) occurring at a dilution ratio of 45, reflecting the competing effects of foam film stability and surface tension.

### 3.2. Mechanical Property

#### 3.2.1. Compressive Strength

**Figure 9:**
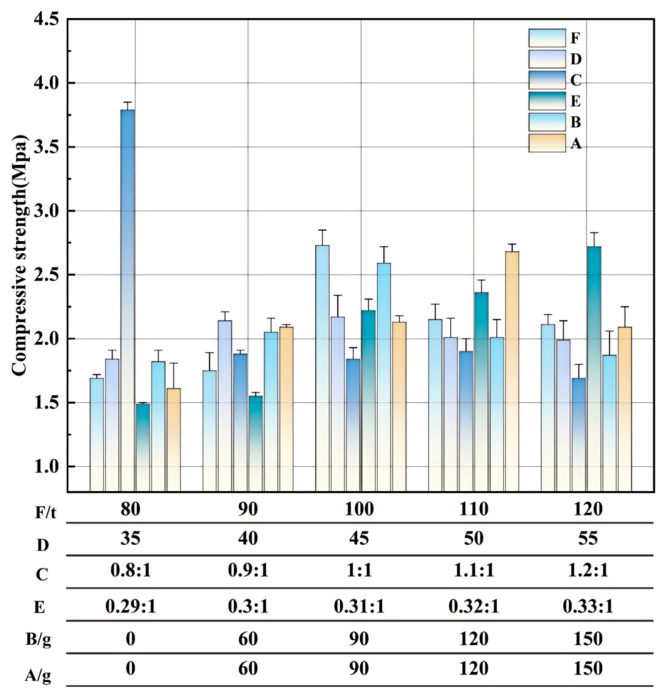
Compressive strength.

**Figure 20:**
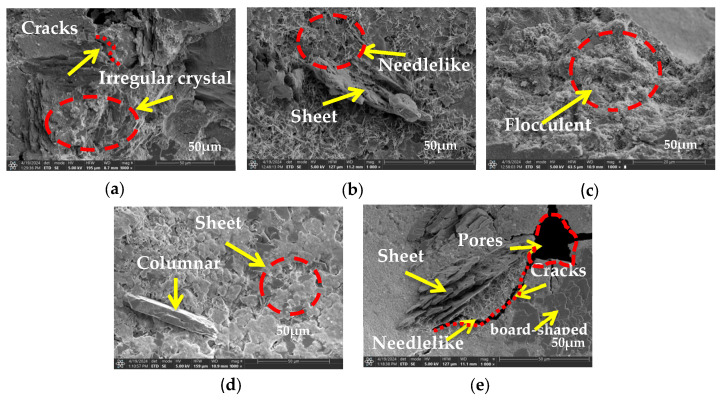
SEM images under different mix proportions. (**a**) H5 Cracks and irregular crystals; (**b**) H9 Sheet and Needlelike; (**c**) H14 flocculent; (**d**) H17 Sheet and columnar; (**e**) H25 Diverse features.

ANOVA results indicated that all tested parameters significantly influenced the compressive strength of SOFS. As shown in Figure 9, Factor A achieved the greatest pore-filling effect at a dosage of 120 g, which corresponded to a denser microstructure in the SEM images presented in Figure 20 (Section 3.5). This optimum likely reflects the balance between pore filling and excessive water adsorption commonly observed in waste-modified systems. However, when the dosage exceeded 120 g, excessive free water adsorption led to the accumulation of unreacted particles, which were visible as clustered and loosely bound regions.

The addition of Factor B improved compactness through bonding between SiO_2_ and the calcium silicate hydrate interface, resulting in a more integrated matrix. Nonetheless, overdosing disrupted particle gradation and introduced localized heterogeneities, as excessive fine aggregate compromises performance, possibly due to increased surface area and water demand.

Increasing Factor C markedly reduced compactness, as reflected by the higher porosity and looser particle arrangements observed in the SEM images. Increased porosity reduces the solid load-bearing area, while discontinuities in the foam skeleton induce stress concentrations. This trend aligns with an exponential decline in strength with increasing foam volume.

Factor D exhibited a nonlinear response, achieving optimal foam monodispersity characterized by a narrow pore size distribution at a dilution ratio of 45. Deviations from this ratio led to bubble coalescence or rupture, forming irregular interconnected voids that concentrate stress.

An optimal balance between hydration product formation and slurry rheology was observed at E = 0.33:1, where SEM revealed uniform hydration products without excessive free water. An adequate water content promotes cement hydration and calcium silicate hydrate gel formation, binding the particles together.

The optimal mixing time for Factor F ranged from 80 to 100 s, during which extended mixing promoted hydration, as evidenced by more complete particle dissolution and gel formation. This improvement results from enhanced foam dispersion and uniform particle distribution. Beyond 100 s, foam rupture and increased pore connectivity were observed, with broken bubble walls and larger connected pores visible in the SEM images.

#### 3.2.2. Splitting Strength

**Figure 10:**
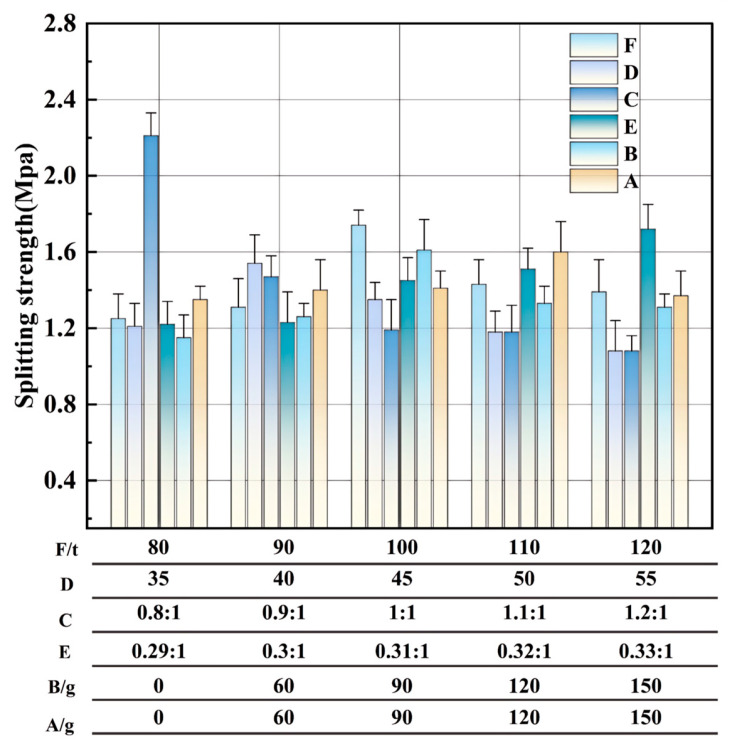
Splitting strength.

As shown in Figure 10, ANOVA results further demonstrated that all tested factors significantly influenced the splitting tensile strength of SOFS. The interfacial occlusion effect was maximized at an A content of 120 g, yielding a splitting tensile strength of 1.60 MPa. At 150 g, excessive free water adsorption led to the accumulation of unconsolidated particles, which inhibited the hydration reaction. The highest interfacial bonding strength (1.61 MPa) was observed at a B content of 90 g, whereas an excessive dosage (150 g) weakened the mechanical properties, corresponding to an 18.6% increase in particle packing looseness. When the C ratio was increased from 0.8:1 to 1.2:1, the splitting tensile strength decreased by 51.1% (from 2.21 to 1.08 MPa), which is primarily attributed to the loss of foam skeleton continuity. Factor D showed an inverted V-shaped trend, with peak foam monodispersity achieved at a dilution ratio of 40, corresponding to a strength of 1.54 MPa. At a ratio of 35, bubble consolidation induced stress concentration, resulting in a strength of 1.21 MPa, whereas at 55, liquid film destabilization reduced the strength to 1.08 MPa. Increasing the E ratio from 0.29:1 to 0.33:1 enhanced the splitting tensile strength by 41% (from 1.22 to 1.72 MPa), an effect attributed to improved crosslinking of the cementitious products. Factor F exhibited a critical threshold within the 80–100 s range. Extending the mixing time improved slurry foam uniformity and hydration, yielding a strength of 1.74 MPa at 100 s—39.2% higher than at 80 s. However, over-mixing (120 s) led to a 20.1% reduction in strength, which may be attributed to defects in the foam structure.

#### 3.2.3. Flexural Strength

**Figure 11:**
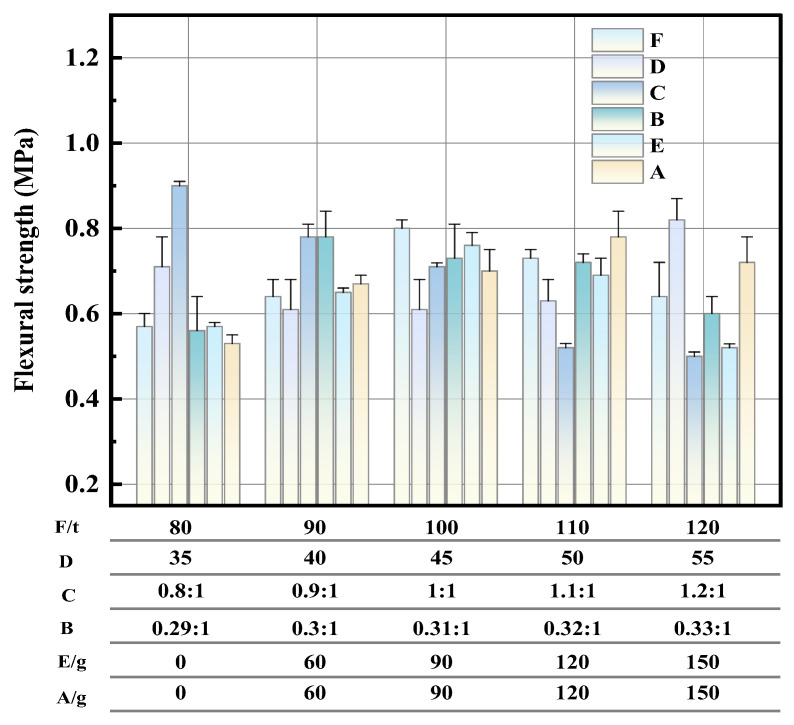
Flexural strength.

ANOVA results indicated that all parameters significantly affected the flexural strength of SOFS. As shown in Figure 11, at an A dosage of 120 g, the flexural strength reached 0.75 MPa, representing a 41.5% increase attributed to shrinkage stress. However, at 150 g, the strength decreased by 4.0%, possibly due to premature coagulation. For Factor B, the optimal interfacial bonding effect was achieved at 60 g, yielding a strength of 0.76 MPa—35.7% higher than the baseline. At an excessive dosage of 150 g, performance declined, corresponding to a 21.1% increase in particle packing looseness. When the C ratio was increased from 0.8:1 to 1.2:1, the strength decreased by 44.4% (from 0.90 MPa to 0.50 MPa), possibly due to reduced foam skeleton density. Factor D showed a nonlinear response: a strength of 0.71 MPa was observed at a dilution ratio of 35×, possibly due to foam aggregation, which increased to 0.82 MPa at 55× as foam homogeneity improved.

Notably, the optimal D value varied across different performance indicators. While the optimal early-age wet density and compressive strength were achieved at a dilution ratio of 45×, the peak flexural strength occurred at 55×. This discrepancy may stem from the differing sensitivity of material properties to foam characteristics. At 45×, the foam likely contributed to higher slurry stability, promoting compactness and early strength development. In contrast, at 55×, the more uniform foam distribution and finer pore size helped alleviate interfacial stress concentration, thereby more effectively enhancing flexural toughness. The optimal E ratio was 0.31:1. At 0.29:1, the strength was 0.57 MPa, possibly due to insufficient cementation, while at 0.33:1, it dropped to 0.52 MPa owing to wall thinning. The optimal F value ranged from 80 to 100 s, within which moderate extension of the mixing time improved bubble homogenization and hydration, yielding a strength of 0.80 MPa at 100 s—40.4% higher than at 80 s. Over-mixing (120 s) led to a 20.0% reduction in strength, possibly due to the breakdown of skeleton continuity. Overall, the flexural strength was governed by a ternary synergistic mechanism involving interfacial bonding, pore distribution, and skeleton integrity.

### 3.3. Optimal Mixture Identification

Range analysis of the influencing factors was performed, and the results are summarized in Table 7. The foam-to-slurry volume ratio (C1: 0.8:1) was identified as the dominant factor contributing to improved mechanical strength. A mixing time of 90 s (F2: 90 s) optimized the wet density and mechanical performance by achieving a balance between foam dispersion and stability. The water-to-solid ratio (E5: 0.33:1) maximized cementing efficiency within the critical range, ensuring an appropriate trade-off between flowability and strength. A foaming agent dilution ratio of 45 (D3: 45×) improved foam monodispersity and reduced the likelihood of pore interconnectivity. The addition of oil sludge residue at 90 g (A3: 90 g) achieved a balance between solid waste utilization and water absorption. Aeolian sand at 120 g (B4: 120 g) enhanced skeleton compactness through siliceous interfacial bonding. The optimal combination, designated H14 (C1F2E5D3A3B4), satisfied the mechanical, construction, and ecological requirements. In addition, four suboptimal formulations (H10, H15, H18, H22) offer adaptable alternatives for various engineering contexts, forming a gradient proportioning system. Accordingly, H14 (A = 90 g, B = 120 g, C = 0.8:1, D = 45, E = 0.33:1, F = 100 s) was identified as the optimal mixture, yielding a 28-day compressive strength of 3.75 MPa, a splitting tensile strength of 2.21 MPa, a flexural strength of 0.9 MPa, a wet density of 940 kg/m^3^, and a flowability of 190 mm.

### 3.4. SOFS Durability

#### 3.4.1. Drying Shrinkage

**Figure 12:**
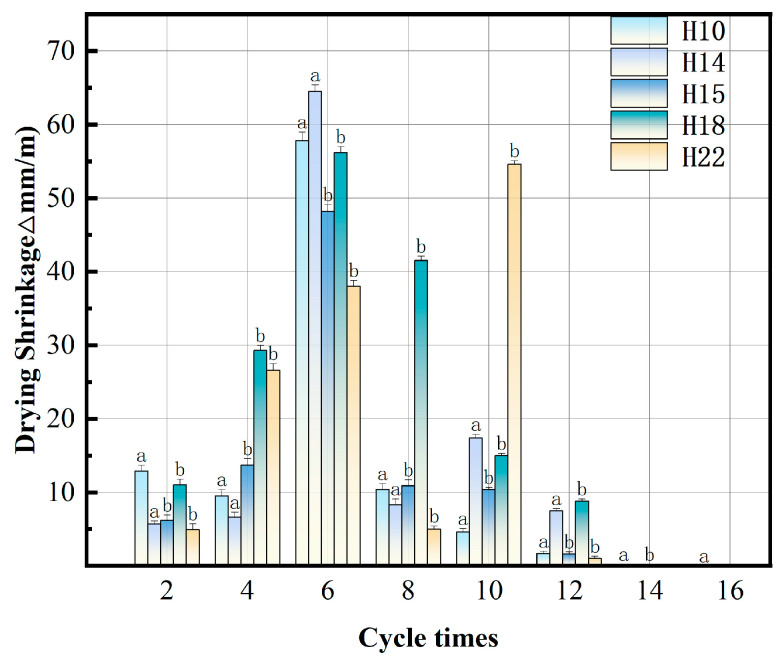
Diagram of Variation in Dry Shrinkage Values. Data are mean ± SEM (n = 3). Different letters denote significant differences between groups within the same cycle (one-way ANOVA with Tukey’s HSD, *p* < 0.05).

These five mixtures were selected to represent a gradient of key compositional parameters: H10 (low residue, high sand), H14 (optimal overall composition), H15 (low water-to-solid ratio, high sand), H18 (high residue, low water-to-solid ratio), and H22 (highest residue, low sand). Figure 12 presents the drying shrinkage values of specimens H10, H14, H15, H18, and H22 after 16 drying cycles. Table 8 and Table 9 presents the content table of influencing factors for five groups of mix proportions.

Figure 12 shows the drying shrinkage values over time. Rapid shrinkage occurred primarily between the fourth and 10th cycles. Statistical analysis (Tukey’s HSD, *p* < 0.05) revealed that within most cycles, the five mixtures fell into two distinct letter groups: ‘a’ (higher shrinkage) and ‘b’ (lower shrinkage). Among the five mix proportions, shrinkage was concentrated around the 6th cycle for most groups. However, in H18 and H22 (group ‘a’), shrinkage continued without a declining trend until after the 10th cycle, highlighting the dominant role of the oil sludge residue content. Specimens H10, H14, and H15 (group ‘b’) exhibited lower drying shrinkage values, faster water dissipation, and earlier structural stabilization. H14 and H15, which were characterized by higher water-to-solid ratios and lower residue-to-sand ratios, developed a fine pore structure and a dense bubble morphology, resulting in high stability and good water retention. In contrast, H18 (group ‘a’), which had a low water-to-solid ratio but a high residue content, formed irregular bubbles and showed reduced water retention; however, its shrinkage increased slightly, possibly due to the presence of the residue. H22 (also group ‘a’) developed larger pores but retained moisture, possibly because of the water absorption capacity of the residue, which left behind residual free water. The consistent letter grouping across cycles confirms that the residue content is the primary factor driving shrinkage, whereas the water-to-solid ratio plays a secondary role.

#### 3.4.2. Dry and Wet Cycle

(1) Appearance

**Figure 13:**
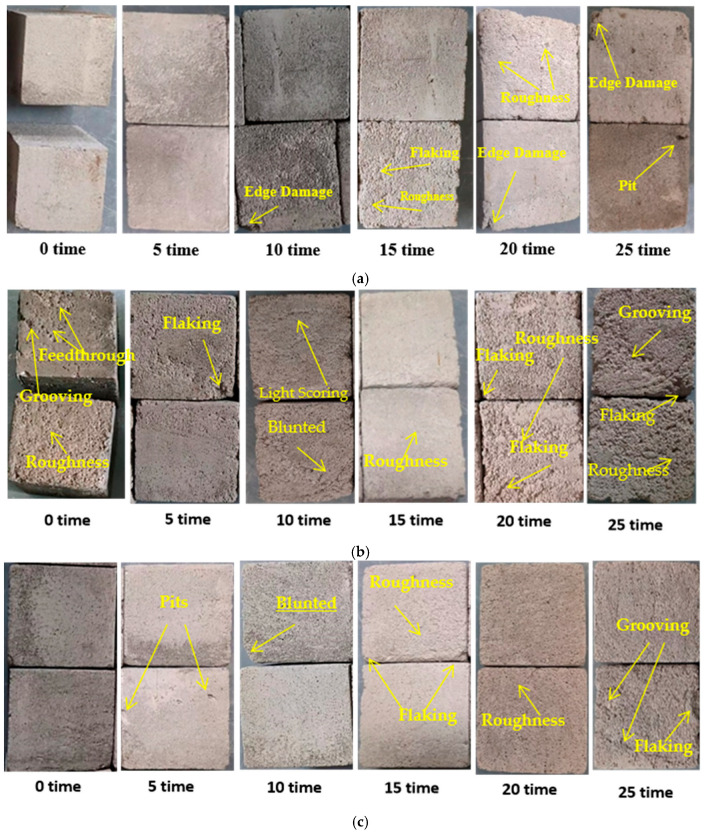

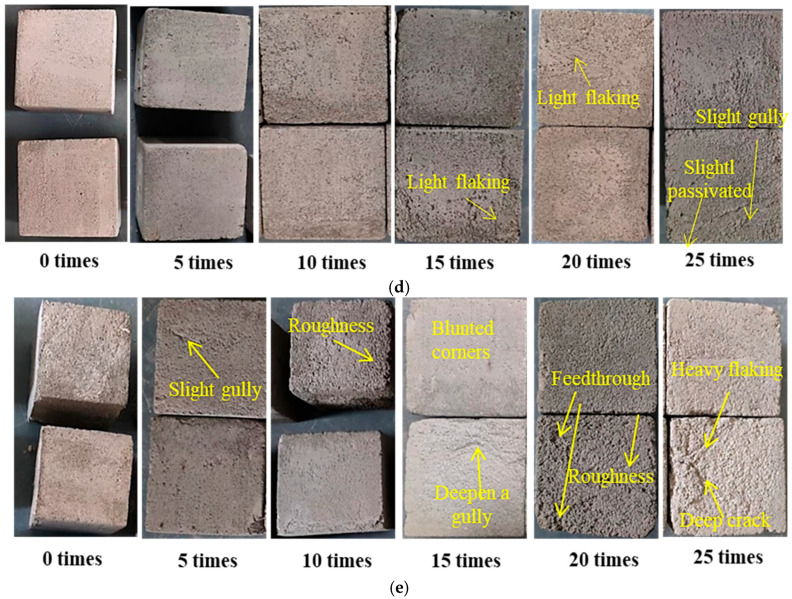
Surface Characteristics. (**a**) H10, (**b**) H14, (**c**) H15, (**d**) H18, (**e**) H22.

Figure 13 illustrates the progression of surface spalling in SOFS specimens with five different mix proportions over 25 wet–dry cycles. As shown in Figure 13a–e, the extent of surface deterioration becomes increasingly severe with more cycles, accompanied by more pronounced edge and corner rounding, regardless of the mix proportion. These observations suggest that alternating wet–dry conditions and thermal effects progressively degrade the internal structure of the specimens, resulting in mass loss and strength reduction. Furthermore, the deterioration patterns vary depending on the mix proportion. As shown in Figure 13a, the H10 specimen exhibited noticeable surface spalling and roughness by the 15th wet–dry cycle. In contrast, the H14 specimen (Figure 13b) showed only slight edge blunting after 25 cycles, with minimal surface degradation and a relatively smooth surface maintained throughout the test.

Figure 13c suggests that after 10 wet–dry cycles, the H15 specimen developed significant spalling and edge damage, accompanied by interconnection and enlargement of the internal foam cavities. After 25 cycles, deep spalling and distinct grooves were observed.

As shown in Figure 13d, the SOFS specimen prepared with H18 initially had an uneven surface with visible through-holes. After 10 wet–dry cycles, these holes enlarged and the surface roughness increased; after 25 cycles, evenly distributed large-diameter pores and fine grooves were observed. However, its overall deterioration was less severe than that of H15.

Figure 13e shows similar observations for another H18-prepared specimen, which initially had surface unevenness and through-holes. The progression of damage followed the same trend: pore enlargement and increased roughness after 10 cycles, and uniform distribution of large pores and grooves after 25 cycles, with the damage remaining less severe than that of H15.

Material performance is governed by five key factors: oil sludge residue content, water-to-solid ratio, foaming agent dilution, foam-to-slurry ratio, and mixing time. A higher water-to-solid ratio enhances flowability and surface finish, while a lower foaming agent dilution improves foam stability. An increased foam-to-slurry ratio reduces density, thereby inhibiting moisture migration and heat transfer. An optimized mixing time ensures proper hydration and uniform foam distribution. Among the mixtures, H10 exhibits excellent durability possibly due to its low residue content, optimal water-to-solid ratio, and homogeneous mixing. H14 and H22 maintain structural integrity through sufficient moisture content and enhanced fluidity; notably, the high foam dilution in H22 may contribute to improved density via controlled foam collapse. In contrast, H15 and H18 show compromised performance possibly due to their high residue content, insufficient moisture, and foam instability, leading to macrovoid formation and reduced resistance to cyclic exposure. Based on the observed damage characteristics in Figure 13, the H14 specimen exhibits the highest resistance to wet–dry cycling among all the evaluated mixes.

(2) Mass Variation Analysis

The structural stability of the specimens under wet–dry cycling varied with the mix proportion, as reflected in the mass loss after 25 cycles: H15 (5.5%), H18 (3.1%), H14 (3.0%), H10 (2.9%), and H22 (2.5%). H22 exhibited the lowest mass loss (2.5%). One-way ANOVA confirmed significant differences among mixtures (F(4,10) = 11.2, *p* < 0.001), and Tukey HSD test showed H22 lost significantly less mass than H15 (5.5%) and H18 (3.1%) (*p* < 0.05), while no significant difference was found among H22, H10 (2.9%), and H14 (3.0%).

**Figure 14:**
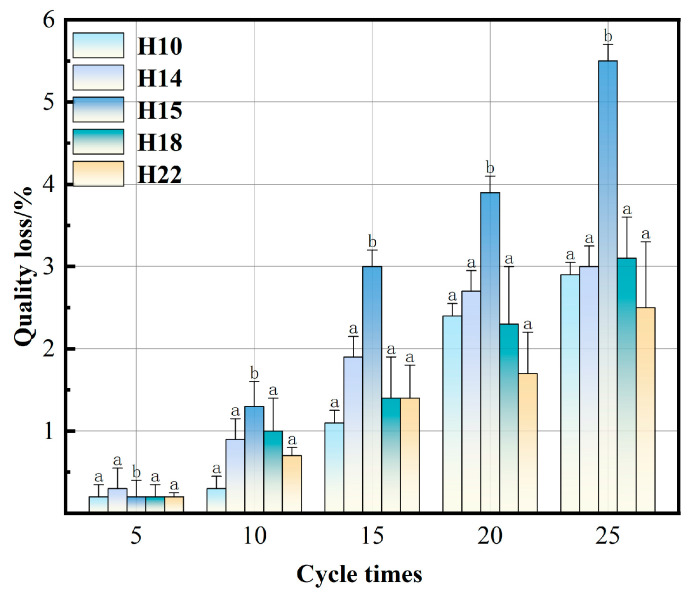
Diagram of Mass Change during Dry–Wet Cycles. Data are mean ± SEM (n = 3). Different letters denote significant differences between groups within the same cycle (one-way ANOVA with Tukey’s HSD, *p* < 0.05).

Critically, H10, H14, and H22 showed better performance possibly due to adequate moisture, thorough hydration, uniform foam distribution, and favorable slurry fluidity, which promoted a homogeneous pore structure. However, the error bars in Figure 14 reveal greater variability for H10 and H14, suggesting less consistent pore formation and hydration control compared to H22, which displayed a tighter data distribution. This implies that while the average performance of H10 and H14 is acceptable, their reproducibility may be an issue.

**Figure 15:**
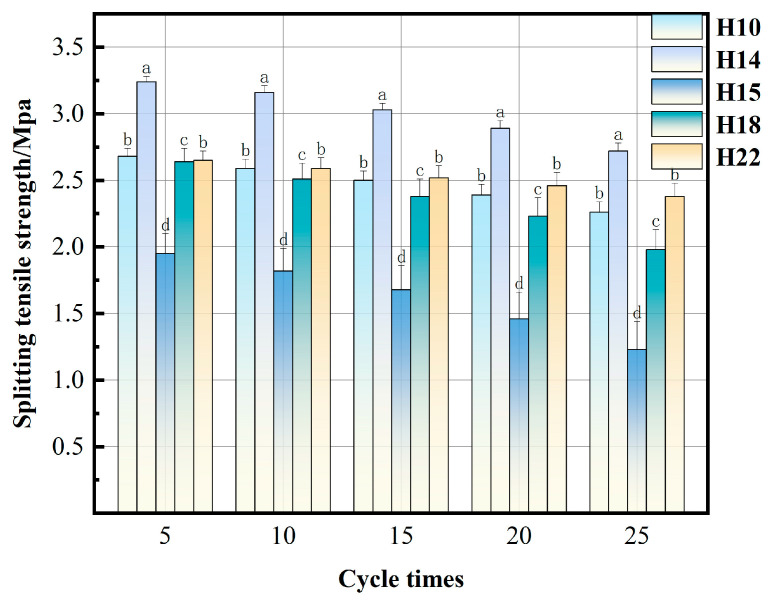
Diagram of Splitting Tensile Strength during Dry–Wet Cycles. Data are mean ± SEM (n = 3). Different letters denote significant differences between groups within the same cycle (one-way ANOVA with Tukey’s HSD, *p* < 0.05).

**Figure 16:**
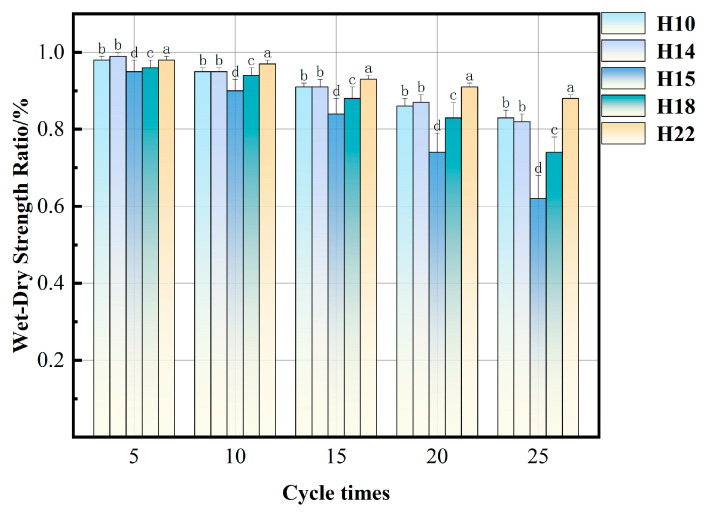
Diagram of Dry–Wet Strength Coefficient. Data are mean ± SEM (n = 3). Different letters denote significant differences between groups within the same cycle (one-way ANOVA with Tukey’s HSD, *p* < 0.05).

In contrast, H15 and H18 suffered from insufficient cement and water, high additive contents, and non-uniform foam generation resulting from a 50× dilution ratio, leading to higher mass loss and wider data scatter. These deficiencies are also evident in Figure 15, where H22 maintained the highest compressive strength with minimal variability, while H15 and H18 showed poor strength retention. Figure 16 further confirms the microstructural densification in H22 compared to the fissures observed in H15 and H18.

For an optimized mix design, the error bar analysis underscores that consistency is as critical as mean performance. H22 emerges as the most robust formulation. H10 and H14 require tighter process control to reduce variability, while H15 and H18 are unsuitable for cyclic exposure. Optimization efforts should prioritize a balanced binder content, an optimal foaming agent dilution ratio, and a workable water content to ensure a uniform pore structure and reproducible durability.

(3) Splitting tensile strength analysis

Figure 15 illustrates the progressive decrease in splitting tensile strength and the corresponding strength coefficient with increasing wet–dry cycles for all mixtures, although the degradation rates varied substantially among the five mixes. After 25 cycles, H14 retained the highest residual strength (2.72 MPa), followed by H22 (2.38 MPa), H10 (2.26 MPa), H18 (1.98 MPa), and H15 (1.23 MPa). The strength loss followed the order H22 (12%) < H10 (17%) < H14 (18%) < H18 (26%) < H15 (45%).

One-way ANOVA confirmed significant differences among mixtures in residual splitting tensile strength after 25 cycles (F(4,10) = 26.4, *p* < 0.001). Tukey HSD post-hoc test showed that H14 (2.72 ± 0.06 MPa) retained significantly higher strength than all other mixtures (*p* < 0.05). H22 and H10 did not differ significantly from each other (*p* > 0.05), while both were significantly higher than H18 and H15 (*p* < 0.05). H15 exhibited the lowest residual strength (1.23 ± 0.21 MPa), significantly worse than all other groups (*p* < 0.05). The variability patterns reflected in the standard deviations were consistent with the mix designs. H14 and H22 exhibited the lowest variability (SD = 0.04–0.10 MPa), indicating excellent reproducibility and microstructural uniformity. In contrast, H15 displayed the widest variation (SD = 0.15–0.21 MPa), reflecting heterogeneous degradation and unstable pore development—a finding consistent with its insufficient water content (0.29:1) and high residue proportion. H18 showed intermediate variability (SD = 0.10–0.15 MPa).

Statistical analysis is consistent with H14 achieved significantly higher residual strength than H22 (*p* < 0.05) while maintaining comparably low variability. The superior performance of H10, H14, and H22 may stem from their adequate moisture content, which facilitates complete hydration and produces fine, uniform pore structures that restrict moisture migration. Conversely, H15 and H18 exhibited insufficient hydration products and higher additive proportions, promoting the formation of large, interconnected pores that accelerated moisture transport and thermal stress damage. H14 thus emerges as the optimal formulation, possibly because it simultaneously maximizes residual strength and minimizes performance dispersion, ensuring reliable long-term durability under cyclic environmental loading.

(4) Dry–wet strength coefficient analysis

As shown in Figure 16, to better illustrate how the strength of the five mixtures evolved over 25 wet–dry cycles, the data were further analyzed and are summarized in Figure 16 At the 25th cycle, the wet–dry strength coefficients for H10, H14, H15, H18, and H22 were 0.83, 0.82, 0.62, 0.74, and 0.88, respectively. A higher coefficient indicates less strength loss in the cycled specimens relative to the control, while a lower value likely reflects more pronounced degradation.

Comparatively, H22 exhibited the highest coefficient (0.88 ± 0.01), which was significantly higher than all other mixtures (one-way ANOVA, F(4,10) = 18.5, *p* < 0.001; Tukey HSD, *p* < 0.05), confirming its superior resistance to cyclic exposure—a finding consistent with its dense microstructure and uniform pore distribution, as discussed earlier. H10 (0.83 ± 0.02) and H14 (0.82 ± 0.02) did not differ significantly from each other, suggesting comparable durability but a slightly greater susceptibility to cyclic effects, likely due to their relatively higher porosity and greater variability in pore structure. H18 showed moderate performance (0.74 ± 0.04), while H15 recorded the lowest coefficient (0.62 ± 0.06), significantly worse than all other groups (*p* < 0.05), underscoring its poor structural integrity under repeated wetting and drying.

H22 showed the smallest variability (SD = 0.01), confirming its high reliability. H15 exhibited both the lowest mean and largest variability (SD = 0.06), rendering it unsuitable for cyclic environments. Future mix optimization should prioritize reducing variability alongside improving mean strength retention. The ranking of H22 > H10 > H14 > H18 > H15 reinforces the idea that mix designs with a balanced binder content, an appropriate foaming agent dilution ratio, and adequate workability lead to better strength retention. For practical mix optimization, H22 serves as a benchmark. H10 and H14 are acceptable alternatives but would benefit from tighter control over mixing uniformity and porosity. In contrast, H15 and H18 are unsuitable for environments subject to cyclic moisture exposure, possibly due to their substantial strength loss. Future design efforts should focus on the synergistic regulation of the binder dosage, foaming agent ratio, and water content to achieve both high strength retention and low performance variability.

#### 3.4.3. Freeze–Thaw Cycles

**Figure 17:**
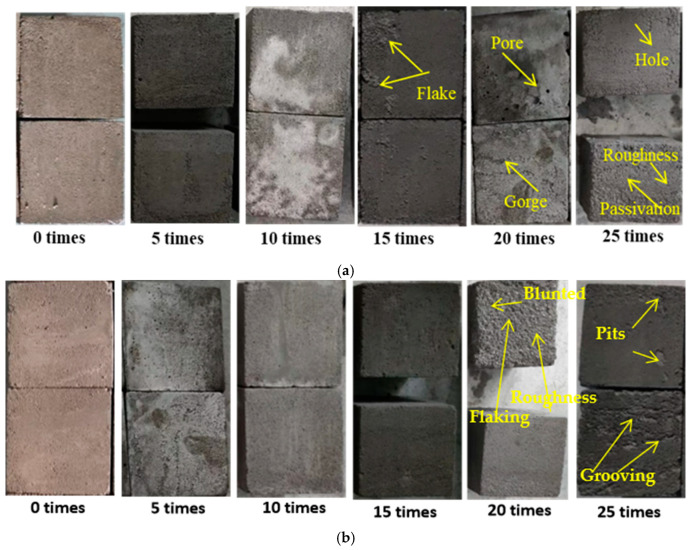

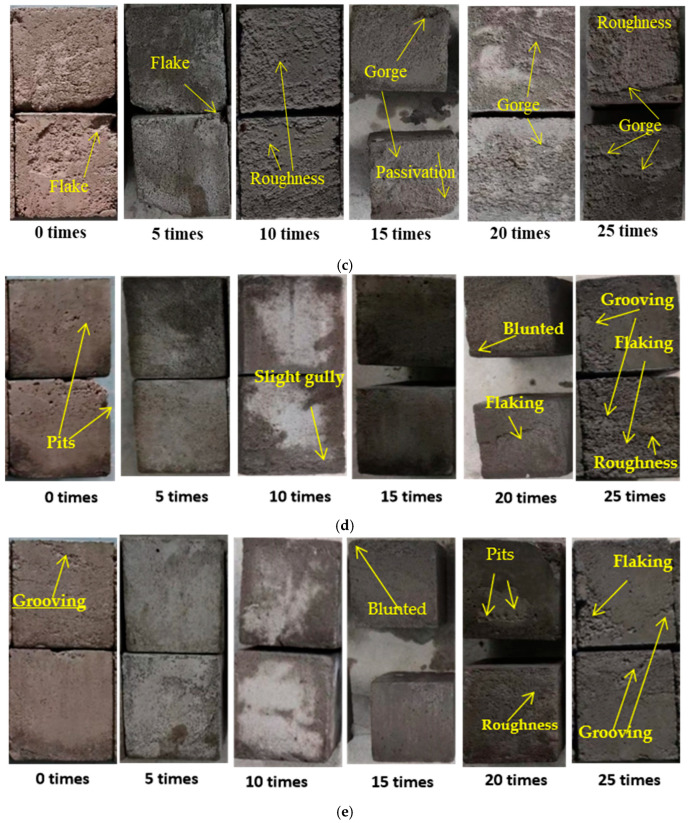
Apparent Process Diagram of Freeze–Thaw Cycles. (**a**) H10, (**b**) H14, (**c**) H15, (**d**) H18, (**e**) H22.

**Figure 18:**
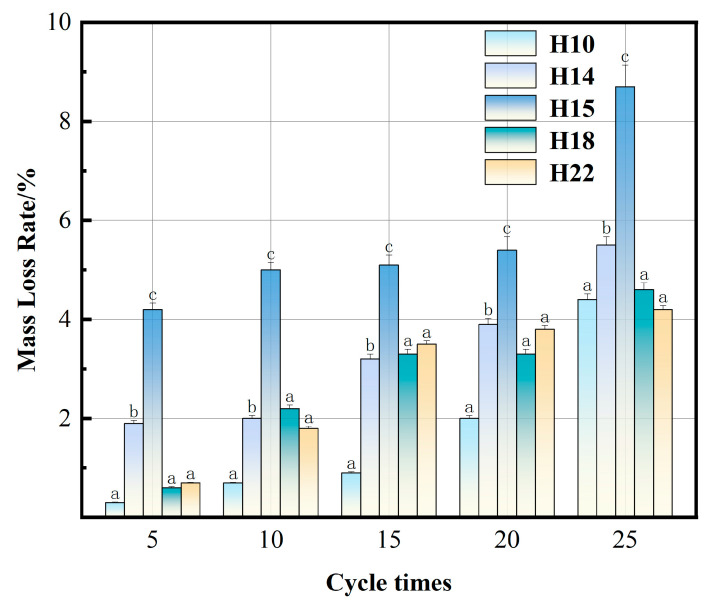
Diagram of Mass Loss Rate of Specimens. Data are mean ± SEM (n = 3). Different letters denote significant differences between groups within the same cycle (one-way ANOVA with Tukey’s HSD, *p* < 0.05).

**Figure 19:**
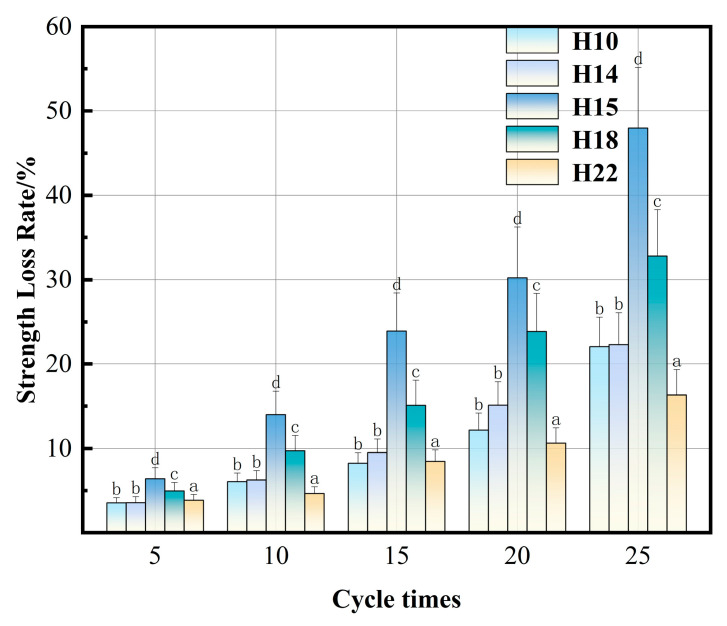
Diagram of Strength Changes during Freeze–Thaw Cycles. Data are mean ± SEM (n = 3). Different letters denote significant differences between groups within the same cycle (one-way ANOVA with Tukey’s HSD, *p* < 0.05).

Figure 17 illustrates the morphological changes under freeze–thaw cycles, Figure 18 presents the mass loss variations, and Figure 19 shows the changes in compressive strength (tested after the completion of the designated freeze–thaw cycles and subsequent conditioning). Through a systematic evaluation of the macroscopic evolution, mass loss rates, and compressive strength degradation, the freeze–thaw resistance of the different SOFS specimens was comprehensively characterized. Statistical analysis (one-way ANOVA followed by Tukey’s HSD post-hoc test) revealed significant differences among the five mixtures in both mass loss (F(4,10) = 18.32, *p* < 0.001) and compressive strength retention (F(4,10) = 22.56, *p* < 0.001) after 25 freeze–thaw cycles.

H10 exhibited visible spalling after 20 cycles, and by the 25th cycle, interconnected pores had led to gully formation and extensive spalling, with a mass loss of 4.4% ± 1.2%. Its strength retention after 25 cycles was 72.3% ± 1.8%. H14 developed minor spalling after 15 cycles and shallow gullies by the 25th cycle, without any corner or edge damage, accompanied by a mass loss of 4.5% ± 0.3%. Its strength retention was 75.1% ± 2.1%. H15, initially loose with through-pores, began spalling after five cycles and formed deep gullies, corresponding to the highest mass loss of 8.7% ± 0.5% (*p* < 0.05 compared to all other groups) and the lowest strength retention of 52.4% ± 2.5% (*p* < 0.05 compared to all other groups). This inferior performance may be attributed to an inadequate water content during mixing, poor workability, and foam coalescence. H18 showed block spalling after 20 cycles and large-area surface spalling with subsurface pore connectivity by the 25th cycle, with a mass loss of 4.6% ± 0.3%. Its strength retention was 65.7% ± 2.2% (*p* < 0.05 compared to all other groups). H22 initially had corner defects, but after 25 cycles it exhibited only surface spalling without major structural failure, achieving a mass loss of 4.2% ± 0.2% (*p* < 0.05 compared to all other groups) and demonstrating the highest strength retention of 78.5% ± 1.9%. H10 and H14 did not differ significantly from each other in either mass loss or strength retention (*p* > 0.05). This performance may be attributed to its dense structure and low porosity, which minimized water ingress and frost heave damage.

The ranking of freeze–thaw resistance differs depending on whether mass loss or strength loss is used as the primary evaluation criterion. Based on mass loss rates (Figure 18), the order from highest to lowest loss is H15 > H18 > H14 > H10 > H22, whereas based on strength retention (Figure 19), the order is H22 > H10 > H14 > H18 > H15. This discrepancy arises possibly because mass loss primarily likely reflects surface deterioration and material shedding, while strength loss indicates internal structural integrity and load-bearing capacity. For engineering applications where structural safety is paramount, strength loss is considered the primary criterion for evaluating freeze–thaw resistance, as it directly governs the serviceability and longevity of the material. Accordingly, considering strength retention, mass loss, admixture utilization, and specimen density, the overall freeze–thaw resistance ranking is H14 > H22 > H10 > H18 > H15.

### 3.5. SOFS Mechanism and Application

#### 3.5.1. Microstructural Mechanism

To analyze the hydration reaction at the ionic level, five mix proportions (H5, H9, H17, H17, and H25) were selected from the 25 groups. The selection was based on the 28-day unconfined compressive strength, the internal cross-sectional condition after the compressive test, and the utilization rates of solid waste and idle resources. Their preparation parameters are shown in the Table 10 below.

Scanning electron microscopy analysis of the H14 specimen revealed a multi-scale structural system underlying its superior mechanical and durability performance, as shown in Figure 20. Micrometer-scale foam cells formed a rigid skeleton through three-dimensional interlocking, featuring dense pore walls between the submillimeter-sized pores. The surfaces of the aeolian sand and oil sludge residue particles were densely covered by hydration products exhibiting flocculent or network-like morphologies, which are characteristic of calcium silicate hydrate gels in cementitious systems, along with plate-like crystals that morphologically resembled calcite.

Notably, nanoscale crystalline phases were observed filling the interfacial pores in H14, as indicated by the red arrows in Figure 20c. Energy-dispersive X-ray spectroscopy analysis revealed that these regions were enriched in aluminum and chlorine, suggesting the possible presence of Al- and Cl-rich phases, potentially resembling aluminum chloride. This phenomenon has rarely been reported in previous studies on foamed concrete. It is tentatively proposed that these nanocrystals formed via a reaction between aluminum ions leached from the oil sludge residue and chloride ions introduced from the foaming agent or mixing water. These in situ-generated nanocrystals further filled the micropores within the calcium silicate hydrate gel network, increasing pore tortuosity and thereby enhancing resistance to moisture ingress and frost heave damage. This mechanism differs from conventional microstructural explanations, which tend to focus solely on calcium silicate hydrate morphology, and may provide new insights into ion-mediated performance optimization in solid waste-based foamed materials. The needle-like morphology observed in some crystalline phases (Figure 20b) is likely characteristic of early-stage calcium silicate hydrate or ettringite, which typically may contribute to early strength development. The co-existence of these needle-like phases with the flocculent C-S-H network and nanoscale AlCl_3_ pore fillers constitutes a multi-stage hydration and densification sequence that progressively refines the pore structure.

Compared to other specimens, H14 exhibited a unique combination of a flocculent-columnar calcium silicate hydrate network, extensive plate-like crystal coverage, and nanoscale pore-filling crystals. This hierarchical microstructure effectively inhibited microcrack propagation, prevented flaky accumulation, and mitigated pore-wall spalling. It should be noted that the present microstructural characterization relies primarily on SEM-EDS analysis. Complementary techniques such as X-ray diffraction (XRD) for crystalline phase identification, thermogravimetric analysis (TG) for hydration product quantification, and Fourier-transform infrared spectroscopy (FTIR) for chemical bonding analysis would provide more definitive evidence for the proposed AlCl_3_ formation mechanism and the composition of the needle-like phases. The absence of these data constitutes a limitation of the current study. Future work will incorporate these techniques to fully validate the microstructural model proposed herein.

#### 3.5.2. Sustainability and Application Prospects

Regarding engineering feasibility, the H14 specimen achieves a wet density of 940 kg/m^3^ and a 28-day compressive strength of 3.75 MPa. These values align with the optimal mix identified in Section 4.3 and satisfy the technical requirements specified in CJJ/T 177-2012 [35] for roadbed fills. According to this standard, materials placed at depths of 0 to 0.8 m beneath pavement surfaces on urban expressways, highways, and primary roads must meet a minimum unit weight of 500 kg/m^3^ and a minimum strength of 0.8 MPa. A comparative analysis with conventional gravel roadbed materials, as detailed in Table 11, confirms the advantageous lightweight characteristics and mechanical performance of the SOFS mixture, thereby demonstrating its practical applicability in roadbed construction.

Beyond technical performance, the proposed SOFS formulation offers notable sustainability benefits. Both aeolian sand and oil sludge pyrolysis residue are locally available in desert regions, which significantly reduces transportation distances and the associated carbon emissions compared to the use of conventional aggregates. The valorization of oil sludge residue addresses a critical environmental issue by converting a waste stream into a valuable construction material, aligning with the principles of the circular economy. Furthermore, the superior durability of the optimal H14 mixture, as evidenced by its resistance to wet–dry and freeze–thaw cycles, implies an extended service life and reduced maintenance interventions, thereby lowering life-cycle resource consumption and the overall environmental impact. While a comprehensive life-cycle assessment and life-cycle cost analysis are beyond the scope of this study, the present findings provide a strong foundation for such future investigations, which would quantitatively validate the environmental and economic advantages of SOFS in desert infrastructure applications.

## 4. Discussion

### 4.1. Synergistic Effects of Aeolian Sand and Oil Sludge Pyrolysis Residue

This study is consistent with the combined use of aeolian sand and oil sludge pyrolysis residue in a foamed lightweight soil, termed SOFS, yielding a material with properties suitable for desert subgrade applications. While the individual feasibility of each material has been established [8,9,10,11,12,13], their synergistic potential remained unexplored. The results show that the optimal mixture, H14, achieves a 28-day compressive strength of 3.75 MPa, a splitting tensile strength of 2.21 MPa, and a flexural strength of 0.9 MPa. These values collectively exceed the requirements for roadbed fills [CJJ/T 177-2012] and represent a significant improvement over systems with a single admixture. For instance, Alharthai et al. [18] reported lower strengths for foamed concrete containing ash from agricultural waste foamed concrete, while the natural fiber-reinforced material developed by Jin et al. [21] did not attain the same combination of strength and durability. The superior performance of H14 may be attributed to the complementary roles of the two residues. Aeolian sand provides a siliceous skeleton that promotes interfacial bonding through C-S-H formation [13], while oil sludge pyrolysis residue contributes reactive phases that participate in hydration and facilitate unique ion-mediated pore-filling effects, as discussed in Section 4.3.

Given the critical importance of compressive strength for subgrade applications, the influence of each experimental factor on this property warrants explicit discussion. In the orthogonal design (Table 5), Factor A represents oil sludge pyrolysis residue content (0–150 g/dm^3^), Factor B aeolian sand content (0–150 g/dm^3^), Factor C the foam-to-slurry volume ratio (0.8:1–1.2:1), Factor D the foaming agent dilution ratio (35–55), Factor E the water-to-solid ratio (0.29:1–0.33:1), and Factor F the mixing time (80–120 s). ANOVA confirmed that all six factors significantly affected compressive strength, with Factor C being the most dominant: increasing foam volume from 0.8:1 to 1.2:1 reduced the load-bearing solid area and introduced stress-concentrating pores, causing strength to drop from 3.75 MPa to 1.08 MPa, a behavior universally reported in foamed concrete [6,14]. Factor E (water-to-solid ratio) exhibited an optimum at 0.33:1—insufficient water limited cement hydration, whereas excess water left capillary voids upon evaporation. Factor A (residue content) improved strength up to 120 g by filling interstitial pores, but overdosing led to unreacted particle accumulation. Factor B (sand content) enhanced compactness through siliceous interfacial bonding up to 90 g, beyond which gradation disruption increased water demand. Factor D (dilution ratio) showed a nonlinear response, peaking at 45, where optimal foam monodispersity produced uniform pores. Factor F (mixing time) improved strength from 80 to 100 s as hydration and foam dispersion progressed, but prolonged mixing damaged the foam skeleton. The resulting 28-day compressive strength of H14 (3.75 MPa) substantially exceeds the 0.8 MPa threshold required by CJJ/T 177-2012 for roadbed fills at a 0–0.8 m depth and compares favorably with fly ash-based foamed concrete of similar density (2.8–3.2 MPa) [1] and recycled powder mixtures (2.5 MPa) [20], confirming that SOFS can reliably meet the structural demands of desert highway subgrades.

### 4.2. Comparison with Existing Foamed Lightweight Soils

The durability of SOFS under cyclic environmental loading is a distinct advantage over conventional foamed lightweight soils. Wu et al. [22] observed that polypropylene fiber-reinforced foamed concrete lost over 25% of its strength after only 20 wet–dry cycles. In contrast, the optimal SOFS mixture H14 retained 82% of its splitting tensile strength after 25 cycles, and H22 retained 88% (wet–dry strength coefficients of 0.82 and 0.88, respectively), demonstrating the effectiveness of the synergistic solid waste modification. This enhanced resistance to wet–dry and freeze–thaw cycles is consistent with the microstructural features described in Section 3.5. A water-to-solid ratio of 0.33:1, as used in H14, proved critical for balancing workability and achieving complete hydration, a finding that supports the conclusions of Yang et al. [34] in their review on sustainable foamed concrete. Furthermore, the stable bubble structure achieved with an optimal foaming agent dilution of 45 effectively suppressed moisture migration and thermal stress development. These factors have been previously identified by Liu et al. [15] and Xiong et al. [4] as key to the stability of foam-stabilized systems.

The freeze-thaw resistance of SOFS also compares favorably with existing materials. Conventional foamed soils are known to suffer progressive internal damage under repeated freezing and thawing possibly due to water ingress and ice crystallization [15]. In H14, the refined pore structure and high pore tortuosity minimized these effects, leading to a mass loss of only 4.5% after 25 freeze-thaw cycles, significantly lower than the 8.7% observed for the poorly optimized H15 mixture. The surface deterioration documented in Figure 13—ranging from superficial roughness in durable mixtures (H14, H22) to deep spalling and groove formation in H15—visually corroborates these quantitative differences and serves as a practical field indicator for assessing in-service subgrade condition. These findings highlight the importance of mix design optimization in achieving durable solid waste-based foamed materials. It should be noted that parallel control specimens were measured only at the final time point to calculate the strength coefficient, not at each cycling interval. The observed strength evolution may therefore partly reflect continued hydration in addition to cyclic damage.

The wet–dry strength coefficients of H22 (0.88) and H14 (0.82) after 25 cycles significantly exceed the 0.65–0.75 range reported for polypropylene fiber-reinforced foamed concrete [22], and the freeze–thaw strength retention of H14 (75.1%) surpasses the 55–65% typical of unmodified foamed concrete after 25 cycles [15]. Among the five evaluated mixtures—H10 (low residue, high sand), H14 (optimal), H15 (low water, high sand), H18 (high residue, low water), and H22 (highest residue, low sand)—those with higher water-to-solid ratios (H14: 0.33:1; H22: 0.32:1) consistently outperformed drier mixes (H15: 0.29:1). This finding corroborates the critical role of sufficient water in achieving complete hydration and a fine, tortuous pore network that restricts moisture ingress and frost damage [34]. The differing optimal formulations for wet–dry resistance (H22) and freeze–thaw resistance (H14) suggest that the two degradation mechanisms impose distinct demands on pore structure, an insight that can guide climate-specific mix design for desert infrastructure.

Several limitations should be acknowledged. The 25-cycle protocol may not fully replicate the coupled effects of thermal cycling, UV exposure, and wind erosion in actual desert environments, and the parallel control specimens were measured only at the final time point, preventing separation of cyclic damage from continued hydration. Despite these caveats, the practical implication is clear: the 30–40% improvement in durability metrics over conventional foamed concrete [15,22] translates directly to extended maintenance intervals and reduced life-cycle costs—a critical advantage in remote desert regions where repair access is logistically difficult.

### 4.3. Microstructural Mechanisms and Their Implications

The microstructural analysis of H14 reveals a hierarchical architecture that distinguishes SOFS from conventional foamed lightweight soils. The flocculent-columnar C-S-H network and extensive plate-like crystals form a robust matrix, while nanoscale crystalline phases filling interfacial pores represent a previously unreported phenomenon. Energy-dispersive X-ray spectroscopy indicated that these regions are enriched in aluminum and chlorine, suggesting the possible formation of AlCl_3_-like nanocrystals. Based on these observations, a plausible formation mechanism may involve the reaction between Al^3+^ leached from the oil sludge pyrolysis residue and Cl^−^ from the foaming agent or mixing water.

This mechanism, in which ions fill the pores, complements the conventional understanding of microstructural development in foamed systems, which has focused primarily on C-S-H morphology [9] and foam stability [15]. The in-situ generated nanocrystals increase pore tortuosity, thereby enhancing resistance to moisture ingress and frost damage. This multi-scale architecture, from the foam-cell skeleton at the micrometer scale to the nanocrystalline phase at the nanometer scale, effectively inhibits microcrack propagation. The absence of the flaky accumulation observed in other mixtures, such as H9, and the lack of pore-wall spalling seen in H25 further confirm the role of this hierarchical structure in maintaining interfacial integrity under environmental loading. This hierarchical architecture directly explains the durability trends observed in Section 3.4. Specimens lacking such multi-scale reinforcement (e.g., H15) exhibited interconnected macro-pores that facilitated moisture migration and freeze–thaw damage, whereas H14’s dense C-S-H network coupled with nanoscale AlCl_3_ pore fillers restricted water ingress paths, directly contributing to its 75.1% strength retention after 25 freeze–thaw cycles. If confirmed by further characterization, the formation of these Al- and Cl-rich phases from waste-derived ions could represent a previously unreported self-densification mechanism in solid waste-based foamed materials.

### 4.4. Sustainability and Practical Applicability

The engineering feasibility of SOFS is supported by its compliance with technical standards. The wet density of 940 kg/m^3^ and the 28-day compressive strength of 3.75 MPa meet the requirements of CJJ/T 177-2012 for roadbed fills placed at depths of 0 to 0.8 m beneath pavement surfaces. A comparative analysis with conventional gravel roadbed materials, presented in Table 11, suggests that SOFS offers advantages in terms of haulage distance, construction equipment demand, maintenance frequency, and resource utilization.

From a sustainability perspective, the use of locally available solid wastes significantly reduces transportation distances and associated carbon emissions. The valorization of oil sludge pyrolysis residue addresses a critical environmental challenge—its long-term stockpiling—by converting a waste stream into a valuable construction material, aligning with circular economy principles [34]. The enhanced durability of H14 implies an extended service life and reduced maintenance interventions, further lowering life-cycle resource consumption and environmental impact [22]. While a comprehensive life-cycle assessment and life-cycle cost analysis are beyond the scope of this study, the present findings provide a foundation for future investigations to quantitatively validate the environmental and economic benefits of SOFS in desert infrastructure applications. The methodological framework developed here, combining orthogonal experimental design with multi-scale characterization, may also be applicable to other solid waste valorization scenarios where synergistic effects between different waste streams are anticipated. The durability data from this study substantiate this sustainability argument: the wet–dry strength coefficient of 0.82–0.88 and freeze–thaw strength retention of 75.1% imply that SOFS can maintain structural integrity with substantially lower maintenance frequency than conventional foamed lightweight soils, which typically exhibit 25–35% higher strength loss under identical cycling conditions [15,22]. This extended service life complements the immediate benefits of local waste utilization, making SOFS a compelling candidate for desert roadbed applications where both material scarcity and environmental sensitivity are acute.

## 5. Conclusions

(1) The optimal SOFS mix proportion (H14) was identified as follows: a foam-to-slurry ratio of 0.8:1, a water-to-solid ratio of 0.33:1, an aeolian sand content of 120 g, an oil sludge residue content of 90 g, a foaming agent dilution of 45, and a mixing time of 100 s. H14 exhibited a flowability of 190 mm, a wet density of 940 kg/m^3^, and 28-day strengths of 3.75 MPa (compressive), 2.21 MPa (splitting tensile), and 0.9 MPa (flexural), thereby satisfying desert roadbed requirements.

(2) Durability assessments under wet–dry and freeze–thaw cycles demonstrated that H14 outperformed the other mixtures with respect to apparent integrity, mass loss, strength retention, and strength coefficient. Its optimal water-to-solid ratio and stable bubble structure effectively inhibited moisture migration and thermal stress damage, enabling the SOFS to maintain high mechanical performance under harsh environmental conditions.

(3) Microstructural analysis revealed that H14 possesses a unique hierarchical structure comprising a flocculent-columnar C-S-H network, high-coverage plate-like crystals, and nanoscale pore-filling phases rich in aluminum and chlorine (potentially AlCl_3_-like). This multi-scale architecture, which has rarely been reported in previous studies, contributed significantly to the material’s mechanical strength and durability by enhancing pore tortuosity and interfacial integrity. Future work should employ complementary microstructural techniques (XRD, TG, FTIR) to further validate the tentatively proposed AlCl_3_ formation mechanism and quantify the hydration products.

(4) The compressive strength of SOFS (3.75 MPa) substantially exceeds the 0.8 MPa requirement for roadbed filling at depths of 0–0.8 m in urban expressways and highways (CJJ/T 177-2012), demonstrating its strong potential for practical application. The synergistic use of aeolian sand and oil sludge residue offers considerable economic and environmental benefits for desert infrastructure development.

## Figures and Tables

**Table 1:** Technical Indicators of Cement.

Specific Surface Area	Compressive Strength (MPa)		Solidification Time (min)	
(m^2^·kg^−1^)	3d	28d	Condensation	Condense
382	27.2	53.1	253	409

**Table 2:** Physical and Chemical Indicators of Aeolian Sand.

Sulfate Content (%)	Natural Moisture Content (%)	Natural Dry Density (g/cm^−3^)	Heaviness (kN/m^−3^)	Mud Content
0.1	1.70	1.66	16.4	1.33

**Table 3:** Grading of aeolian sand and oil sludge pyrolysis residues.

Particle Size/mm	Mass Fraction of Aeolian Sand/%	Oil Sludge Pyrolysis Residue Mass Fraction/%
≥7~10	0	0.92
≥5~7	0.11	1.62
≥3~5	0.35	3.67
≥1~3	2.62	1.35
≥0.5~1	10.20	2.67
≥0.25~0.5	16.21	2.13
≥0.075~0.25	69.73	60.05
0~0.075	0.78	27.59

**Table 4:** Foaming agent type.

Dilution Factor	35	40	45	50	55
Type					
Densities (kg/m^3^)	46.9	49.3	52.6	54.5	56.2
water secretion rate/%	66.6	68.8	70.6	72.3	75.6
Foaming power	23.22	22.09	20.70	19.98	19.38

**Table 5:** Orthogonal test factor level table.

A	B	C	D	E	F
0	0	0.8:1	35	0.29	80
60	60	0.9:1	40	0.3	90
90	90	1:1	45	0.31	100
120	120	1.1:1	50	0.32	110
150	150	1.2:1	55	0.33	120

**Table 6:** Orthogonal test mix ratio design table.

Factor	A	B	C	D	E	F
Serial Number						
H1	0	0	0.8	35	0.29	80
H2	0	60	0.9	40	0.30	90
H3	0	90	1.0	45	0.31	100
H4	0	120	1.1	50	0.32	110
H5	0	150	1.2	55	0.33	120
H6	60	0	0.9	45	0.32	120
H7	60	60	1.0	50	0.33	80
H8	60	90	1.1	55	0.29	90
H9	60	120	1.2	35	0.30	100
H10	60	150	0.8	40	0.31	110
H11	90	0	1.0	55	0.30	110
H12	90	60	1.1	35	0.31	120
H13	90	90	1.2	40	0.32	80
H14	90	120	0.8	45	0.33	90
H15	90	150	0.9	50	0.29	100
H16	120	0	1.1	40	0.33	100
H17	120	60	1.2	45	0.29	110
H18	120	90	0.8	50	0.30	120
H19	120	120	0.9	55	0.31	80
H20	120	150	1.0	35	0.32	90
H21	150	0	1.2	50	0.31	90
H22	150	60	0.8	55	0.32	100
H23	150	90	0.9	35	0.33	110
H24	150	120	1.0	40	0.29	120
H25	150	150	1.1	45	0.30	80

**Table 7:** Fluidity variance analysis.

Variable	SS	DF	MS	F	*p*	Critical Value	Signif-
OPRC	1006.18	4	251.545	2.375	0.080	F0.01(4,25) = 4.177 F0.05(4,25) = 2.76 F0.1(4,25) = 2.18	(*)
ASC	3960.43	4	990.108	9.347	0.00093 **		**
FSVR	345.88	4	86.47	0.816	0.526		
FADR	436.33	4	109.082	1.03	0.411		
W-SR	2111.63	4	527.908	4.984	0.004 **		**
Mixing Time	453.53	4	113.382	1.07			
Error	2648.13	25	105.925				

Note: OPRC: Oil Sludge Pyrolysis Residue Content. ASC: Aeolian Sand Content. FSVR: Foam Slurry Volume Ratio. FADR: Foaming Agent Dilution Ratio. W-SR: Water-Solid Ratio. **—highly significant; (*)—some influence; blank—no significant effect.

**Table 8:** Analysis Table of Fluidity Range and Strength Indices.

Factor	A	B	C	D	E	F	Optimal Solution
Range Analysis							
Flowability	6.9	20.9	8.4	15.5	12.7	8.5	B_5_D_1_E_5_F_1_C_1_A_5_
Wet density	162.5	54.0	305.5	137.6	114.5	243.0	C_5_F_2_A_1_D_2_E_2_B_2_
Compressive	1.07	0.77	2.10	0.33	1.23	1.04	C_1_E_5_A_4_F_3_B_3_D_3_
Splitting	0.55	0.38	1.37	0.64	0.52	0.74	C_1_F_2_D_3_A_3_E_4_B_2_
Flexural	0.19	0.22	0.38	0.26	0.23	0.29	C_1_F_3_D_2_E_3_B_2_A_4_

**Table 9:** Mixture parameters and test conditions.

Mix Ratio	Influencing Factors					
	Oil Sludge	Aeolian Sand	Water-to-Solid Ratio	Foam-to-Slurry Volume Ratio	Foaming Agent Dilution Ratio	Mixing Time
H10	60	150	0.31	0.8:1	40	110
H14	90	120	0.33	0.8:1	45	90
H15	90	150	0.29	0.8:1	50	100
H18	120	90	0.30	0.8:1	50	120
H22	150	60	0.32	0.8:1	55	100

Note: H14 is the optimal mixture, with 28-day compressive strength of 3.75 MPa, splitting tensile strength of 2.21 MPa, and flexural strength of 0.9 MPa.

**Table 10:** Mix proportions of the test samples.

Sample No.	Oil Sludge Pyrolysis Residue (g)	Aeolian Sand (g)	Foam-to-Slurry Volume Ratio	Dilution Ratio	Water-to-Solid Ratio	Mixing Time (s)
H5	0	150	1.2:1	55	0.33:1	120
H9	60	120	1.2:1	35	0.30:1	100
H14	120	90	0.8:1	45	0.33:1	100
H17	120	60	1.2:1	45	0.29:1	110
H25	150	150	1.1:1	45	0.30:1	80

**Table 11:** Comparison of conventional roadbed materials and foam lightweight soil base in terms of cost-related factors.

Cost	Conventional Roadbed Materials	Foam Lightweight Soil Base
Haulage	**High** (long distance traveled)	**Low** (solid waste utilization)
Construction	**High** (high demand for heavy machinery)	**Low** (lightweight self-leveling)
Maintenance	**High** (frequent settlement/cracking)	**Low** (High durability)
Resources	**High** (aggregate mining)	**Low** (solid waste utilization)

## Data Availability

The raw data supporting the conclusions of this article will be made available by the authors on request.
